# Parasitic gastritis in wild sunda pangolins (*Manis Javanica*), Singapore

**DOI:** 10.1007/s00436-025-08567-0

**Published:** 2025-10-01

**Authors:** Shin Min Chong, Kei Chloe Tan, Delia Hwee Hoon Chua, Liesbeth Frias, Chiharu Okumura

**Affiliations:** 1 Mandai Wildlife Group, 80 Mandai Lake Road, Singapore, 729826 Singapore; 2 School of Animal and Veterinary Sciences, University of Adelaide, Roseworthy Campus, South Australia, Australia; 3 Graduate School of Agricultural Science, Tohoku University, Sendai, 980-0845 Japan; 4 Department of Infectious Diseases and Public Health, Jockey Club College of Veterinary Medicine and Life Sciences, City University of Hong Kong, Hong Kong SAR, China

**Keywords:** *Gendrespirura*, Parasites, Wildlife health, Wildlife conservation, Critically-endangered species

## Abstract

Gastrointestinal parasites have been reported in wild Sunda pangolins (*Manis javanica*), but the clinical significance of such detections is often unknown. This report describes pathological lesions in the stomachs of two dead wild Sunda pangolins caused by *Gendrespirura* sp.. Gross and histopathological examination showed gastritis and hyperkeratosis in infected animals. Morphological identification confirmed the presence of *Gendrespirura* sp., and molecular analysis revealed a close phylogenetic relationship with the genus *Gendrespirura,* supporting placement within the family Habronematidae. This report highlights the pathological significance of parasite infections in wild pangolins in Singapore, further advancing our understanding of the natural biology and parasitology of wild Sunda pangolins, and the effects of these infections on the health and conservation of this critically endangered species.

**Supplementary Information:**

The online version contains supplementary material available at 10.1007/s00436-025-08567-0.

## Introduction

The Sunda pangolin (*Manis javanica*) is one of the four pangolin species that can be found in Asia. It inhabits a diverse landscape across Southeast Asia, where its range includes Thailand, Laos, Vietnam, Cambodia, Peninsular Malaysia, Singapore, Sumatra, Java, adjacent islands, and Borneo (Challender et al. 2019). Unfortunately, the Sunda pangolin faces severe threats, including illegal poaching and trafficking in Southeast Asia driven by the demand for their scales and meat in East Asia, leading to significant population declines and classification as Critically Endangered (CR) by the International Union of Conservation of Nature (IUCN) Red List (Challender et al. 2019; Huang et al. 2021).

In Singapore, the Sunda pangolin is also listed as threatened but faces a different and unique threat to its population; road traffic collisions (Aziz et al. 2024). These animals reside in primary and secondary forests, and forage for ants or termites in soil or rotting vegetation by digging with their powerful claws. Many pangolins stray out of their natural habits and end up on busy urban roads, resulting in fatal road traffic collisions. Other threats such as infectious diseases have not been reported in local pangolins, although endoparasites in Sunda pangolins from other range countries have been reported (Mohapatra et al. 2016; Barton et al. 2022). In this report, we present the pathological findings from two Sunda pangolins in Singapore affected by gastric parasites, along with the morphological and molecular identification of those parasites.

## Materials and methods

### Post-mortem examination and sampling

All rescued and deceased wild pangolins reported by members of the public or wildlife rescue groups are brought to the Singapore Zoo, Mandai Wildlife Reserve, for veterinary attention. Dead pangolins are subject to a post-mortem examination by a veterinary pathologist. Two dead Sunda pangolins submitted to the Singapore Zoo were examined (cases 1 and 2), and live and dead nematode specimens from the stomach of case 2 were collected into 70% ethanol for microscopic examination and molecular identification. Representative samples of the stomach from cases 1 and 2 were taken and fixed in 10% buffered formalin saline (BFS), then processed routinely by sectioning them at 4 µm thickness and staining them with hematoxylin and eosin (H&E) for histology examination for pathological lesions. Specimens in 70% ethanol were deposited at the Zoological Reference Collection of the Lee Kong Chian Natural History Museum of the National University of Singapore (Accession no. ZRC.NMT.0033).

### Parasite identification

#### Morphological identification

Four parasite specimens preserved in 70% ethanol were examined under a stereoscope (Olympus SZX7, Singapore) and light microscope (Olympus BX 43, Singapore), utilizing a mounted digital camera (Olympus DP22, Singapore). Measurements of morphological structures of adult worms were obtained using the software ZEISS Labscope v4.4 on a ZEISS Primostar 3 microscope at × 100 and × 400 magnifications.

#### Molecular identification

DNA extraction was performed using QIAGEN QIAamp® DNA Mini Kit (Qiagen, Hilden, Germany), following the manufacturer’s instructions. Two primer sets targeting the mitochondrial cytochrome c oxidase I (COX1) gene, and a set of primer targeting the 18S small subunit rRNA (18S rRNA) gene were tested. The primer sequences and size of the amplicons are listed in Table 1. PCR conditions were followed, according to the cited references.

**Table 1:** Primer sequence details used for molecular identification of nematodes retrieved from a wild Sunda pangolin (case 2)

Target	Primer Name	Primer Sequence (5’ to 3’)	Product Size (bp)	Reference
COX1	NTF	TGATTGGTGGTTTTGGTAA	649	Barton et al. 2022
	NTR	ATAAG TACGAGTATCAATATC		
COX1	JB3	TTTTTTGGGCATCCTGAGGTTTAT	441	Li et al. 2024
	JB4.5	TAAAGAAAGAACATAATGAAAATG		
18S rRNA	Nem_18S_F	CGCGAATRGCTCATTACAACAGC	~ 900	Floyd et al. 2005
	Nem_18S_R	GGGCGGTATCTGATCGCC		

The amplified DNA was submitted to an external laboratory for Sanger sequencing and ambiguous ends were trimmed followed by consensus sequences aligned using the forward and reverse sequencing data in BioEdit, with the integrated ClustalW tool. The consensus sequences obtained were compared with existing sequences in BLASTn (Altschul et al. 1997) to assess preliminary identity. COX1 sequences generated using the primer set from Barton et al. (2022) were used in the main phylogenetic analysis. To ensure sufficient alignment length and resolution, only GenBank reference sequences (Clark et al. 2016) with at least 300 bp overlap were retained. The final dataset included 35 sequences from species in the superfamily Habronematoidea, and one outgroup sequence belonging to *Onchocerca volvulus.* Sequences were aligned using MAFFT v7 with the G-INS-i algorithm (Katoh and Standley 2013) and a maximum likelihood (ML) tree was generated using IQ-TREE v2.2.0 (Minh et al. 2020) under the best-fit model (GTR + F + I + G4), selected by ModelFinder (Kalyaanamoorthy et al. 2017). The tree was visualized and edited in FigTree v1.4.4 (Rambaut 2018) and Inkscape (Inkscape Project 2024).

To complement the main analysis, additional COX1 and 18S sequences were amplified using primer sets from Li et al. (2024) and analyzed separately due to minimal overlap with the Barton COX1 region. These datasets were compared against a broader set of Spiruromorpha reference sequences in GenBank, and phylogenetic trees were generated using the same ML pipeline as above. The reference sequences used are listed in Tables S2 (18S) and S3 (COX1).

## Results

### Post-mortem examination

**Fig. 1:**
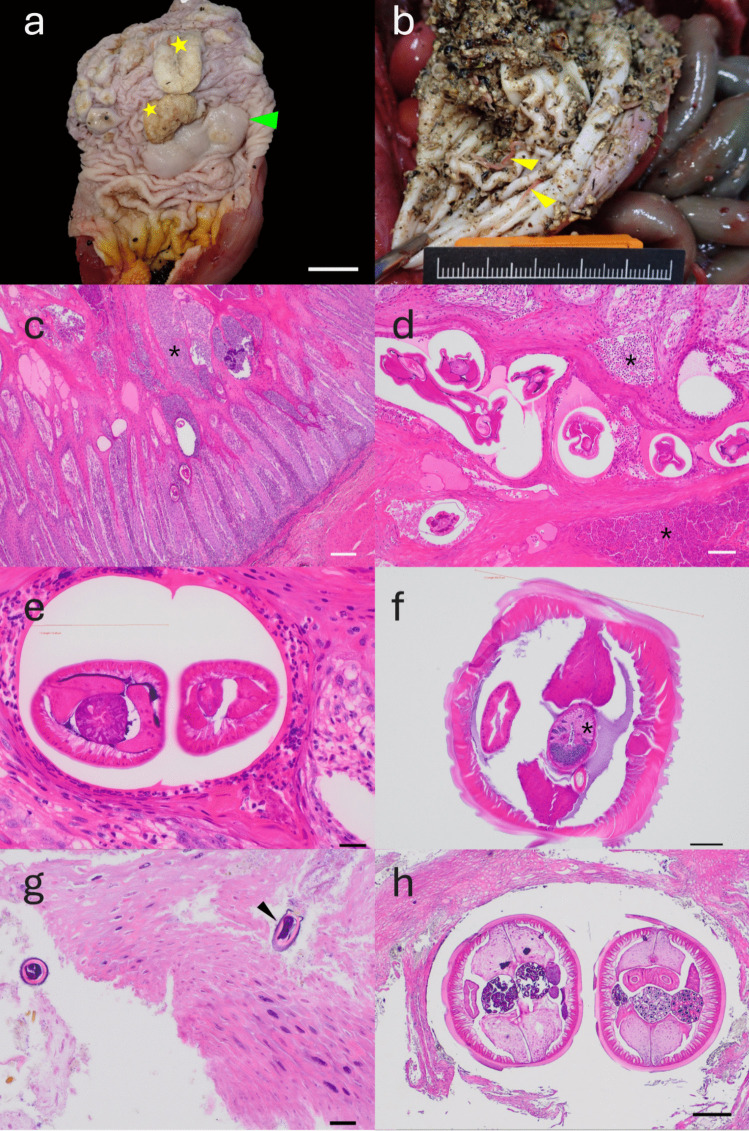
Gross photographs and photomicrographs of two wild Sunda pangolins (*Manis javanica*) from Singapore with parasitic gastritis (**a**) Case 1, cornified mucosa of the stomach is raised, thickened, nodular and irregular (stars), affecting up to 10–40% of the stomach. Normal oxyntic gland (arrowhead) (scale bar = 2 cm) (**b**) Case 2, large numbers of nematodes (> 50) measuring 15–20 mm (arrowhead) in the stomach lumen and gastric contents (scale bar = 5 cm) (**c**) Case 1, moderate to marked hyperkeratosis and embedded parasite larvae with diameters ranging from 45–69 µm, with neutrophilic inflammation (*) (scale bar = 200 µm) (**d**) Case 1, magnified view of larvae shows thick cuticle, coelomyarian musculature, pseudocoelom and digestive tract. Surrounding neutrophilic inflammation (*) (scale bar = 50 µm) (**e**) Case 1, magnified view of embedded parasite larvae surrounded by neutrophilic inflammation. Diameter of left specimen is 115.2 µm (scale bar = 20 µm) (**f**) Case 1, adult parasite in stomach lumen, diameter 395.8 µm, male gonad (*) (scale bar = 50 µm) (**g**) Case 2, nematode eggs measuring 25–36 µm, with thick capsule, embryonated larvae and barrel-like cap (arrowhead) (scale bar = 20 µm) (**h**) Case 2, transverse sections of adult nematode (scale bar = 200 µm)

Post-mortem gross examination showed that both animals were male. Case 1 weighed 9.4 kg with a body condition score of 3/5 (Chou et al. 2021), while case 2 weighed 7.35 kg and also had a body condition score of 3/5 (Chou et al. 2021). Both animals died from high-impact blunt force traumatic injuries consistent with those of a vehicular collision. Unrelated to the traumatic injuries, both pangolins had pathological lesions in the stomach with the presence of nematodes in the lumen and embedded within the gastric mucosa. In case 1, the cornified mucosa was raised, thickened, nodular and irregular, affecting up to 10–40% of the entire stomach (Fig. 1a). Low numbers (< 5) of nematodes were present in the stomach lumen or embedded in the mucosa. In case 2, large numbers (> 50) of nematodes measuring 15–20 mm were present in the stomach lumen and gastric contents (Fig. 1b). Histopathological examination of H&E-stained stomach sections from case 1 showed transverse sections of larvae measuring 45–69 µm across embedded in the epithelium with large numbers of neutrophils surrounding and infiltrating the mucosa, forming small pustules with moderate to marked hyperkeratosis (Fig. 1c, d). These parasites had a thick cuticle, coelomyarian musculature, pseudocoelom, digestive tract, and prominent lateral cords. Larger parasites embedded in the epithelium and in the lumen also had thick cuticle, coelomyarian musculature, pseudocoelom, digestive tract, prominent lateral cords and gonads (Fig. 1e, f). In case 2, parasite eggs measuring 25–36 µm across with a thick capsule and embryonated larvae were embedded within the hyperkeratotic and hyperplastic stratum corneum and mucosa. Some eggs had barrel-like caps (Fig. 1g). Higher magnification showed that the embedded adult parasites within cysts in the stratum corneum had thick cuticle, coelomyarian musculature, pseudocoelom, digestive tract, lateral cords and reproductive organs (Fig. 1h).

### Morphological examination

**Fig. 2:**
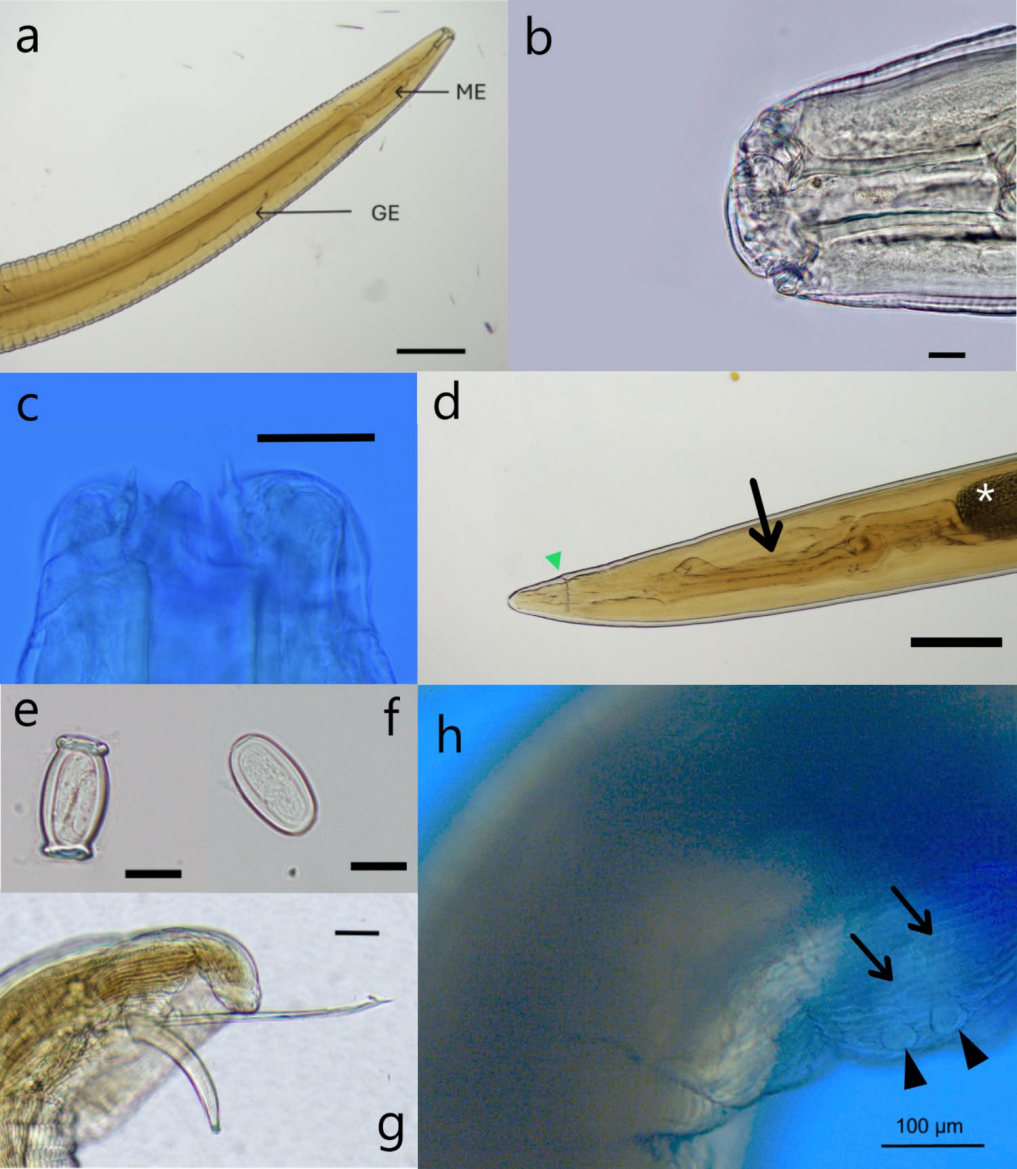
Photomicrographs of *Gendrespirura* sp. retrieved from a wild Sunda pangolin (*Manis javanica*) causing gastritis (case 2). (**a**) Female, anterior end showing shorter anterior muscular esophagus (ME) and wider posterior glandular esophagus (GE) (scale bar = 500 µm) (**b**) Female, anterior end, mouth, elongated cylindrical buccal cavity and pseudolabia (scale bar = 20 µm) (**c**) Female, anterior end, showing teeth on lateral pseudolabia (scale bar = 50 µm) (**d**) Female, posterior end, gradually tapered body shape, bluntly rounded showing anus (arrowhead), intestinal (arrow) and ovarian structures (*) (scale bar = 500 µm) (**e**) Eggs extracted from one female specimen show mature eggs with thick walls and polar ends with raised barrel-like caps (**f**) Immature eggs from the same female specimen (**e–f** scale bar = 20 µm) **(g)** Male, posterior end, spiral tail, paired copulatory spicules (scale bar = 100 µm) **(h)** Male, posterior end, pre-cloacal papillae (arrowheads) and ridges (arrows) on the ventral surface (scale bar = 100 µm)

Microscopic examination of one female and one male specimen identified the parasite as *Gendrespirura* sp. General. Medium-sized body tapering gradually at anterior and posterior extremities. Cuticle transversely striated along the entire body length, with no lateral alae present (Fig. 2a). Cephalic region features two large lateral pseudolabia, each equipped with three teeth on the inner surface, where the central tooth is more prominent than the lateral teeth (Fig. 2b, c); one pair lateral amphids. Buccal capsule cylindrical, well-developed, and sclerotized. Esophagus long, comprising a shorter, narrow muscular portion and a longer, wider glandular portion. Female (one specimen). Vulva located in anterior part of body. Two sections of uteri suggest a didelphic structure (Fig. 1h). Tail curved with blunt rounded end (Fig. 2d). Mature eggs small, featuring a thick wall with polar ends that have a thickened cuticle, forming raised hoops that create a barrel-like appearance (Fig. 2e). Immature eggs lack thickened hoops (Fig. 2f). Detailed measurements are indicated in Table 2. Male (one specimen). Posterior extremity coiled, caudal alae developed and symmetrical. Post-anal papillae, not all observed in current specimens. Spicules uneven, left spicule long and slender, right spicule short and stout (Fig. 2g). Ratio of length of left spicule to right spicule 4.88. Gubernaculum present. Ridges on ventral surface of nematode, anterior to cloacal region (Fig. 2h). Detailed measurements are indicated in Table 2.

**Table 2:** Measurements of female and male *Gendrespirura* sp. collected from Sunda pangolins from Singapore compared to previous reports of parasite specimens collected from various pangolins in Asia, according to Barton et al. 2022. Measurements are presented in micrometers

original identification	*Gendrespirura* sp.	*Gendrespirura cf. zschokkei*	Habronema hamospiculatum*	*Gendrespirura zschokkei*	*Gendrespirura kwangtungensis*	*Gendrespirura* sp.
host	*M. javanica*	*M. javanica*	*M. javanica*	*M. crassicaudata*	*M. pentadacyla*	*M. crassicaudata*
location	Singapore	Malaysia	Vietnam	Sri Lanka	China	Sri Lanka
reference	this study	Barton et al. 2022	Hsü 1932	Meyer 1896	Kou 1958	Von Linstow 1906
female specimen						
body length	19,107	23,662.5 (21,950–25,375)	15,700–23,800	32,000 (25,000–35,000)	25,810–29,510	32,000
body width	740	453.1 (337.5–550)	500–800	1300	770–885	950
no. pairs of teeth on pseudolabia	3	3	3	–	7	–
nerve ring to anterior end	387	400 (350–450)	260–310	–	330–370	–
cervical papillae to anterior end	231	292.5 (230–390)	–	–	265–306	–
excretory pore to anterior end	–	540 (250–900)	–	–	–	–
vestibule length	186	120 (105–135)	150–180	114	–	200
esophagus length	3,239	3,940 (2,430–5,100)	3,800–3,920	6,400 (5,000–7,000)	4,500–5,370	9,143
vulva to anterior end	6,033	6,243.3 (5,180–7,850)	5,380	8,000	7,350–7,750	18,667
egg length x width	Mature: 44.6 (43–46.2) × 24.5 (23.1–26.8) Immature: 39.1 (37.3–41.2) × 22.6	Mature: 46.7 (45–47.5) × 25.4 (25–26.25) Immature: 35.6 (32.5–37.5) × 22.8 (21.25 × 25)	42–43 × 24–26	43 × 23–24	46–47 × 26–27	47 × 29
tail length	298	233.8 (155–305)	280–440	330–380	–	485
male specimen						
body length	13,731	13,375	11,400–16,100	21,000 (19,000–24,000)	18,250–22,380	25,000
body width	486	500	380–590	1100	664–758	710
no. pairs of teeth on pseudolabia	3	3	3	–	7	–
nerve ring to anterior end	231	–	230–250	–	324–367	–
cervical papillae to anterior end	–	230	–	–	265–278	–
excretory pore to anterior end	–	–	–	–	–	–
buccal cavity length	139	130	132–145	114	–	200
esophagus length	3,128	3,800	3,240–3,900	4,200 (3,800–4,800)	3,337–4,651	8333
left spicule length	3,290	3,400	3,170–3,450	4,700–5,300	3,010–3,230	3740
right spicule length	674	725	550–620	1,800–2,000	650–710	570
gubernaculum length		110	70	–	–	–
tail length	321	370	360–450	490	–	481

* *Gendrespirura* was originally reported as *Habronema hamospiculatum* Neveu-Lemaire, 1927

### Molecular identification

**Fig. 3:**
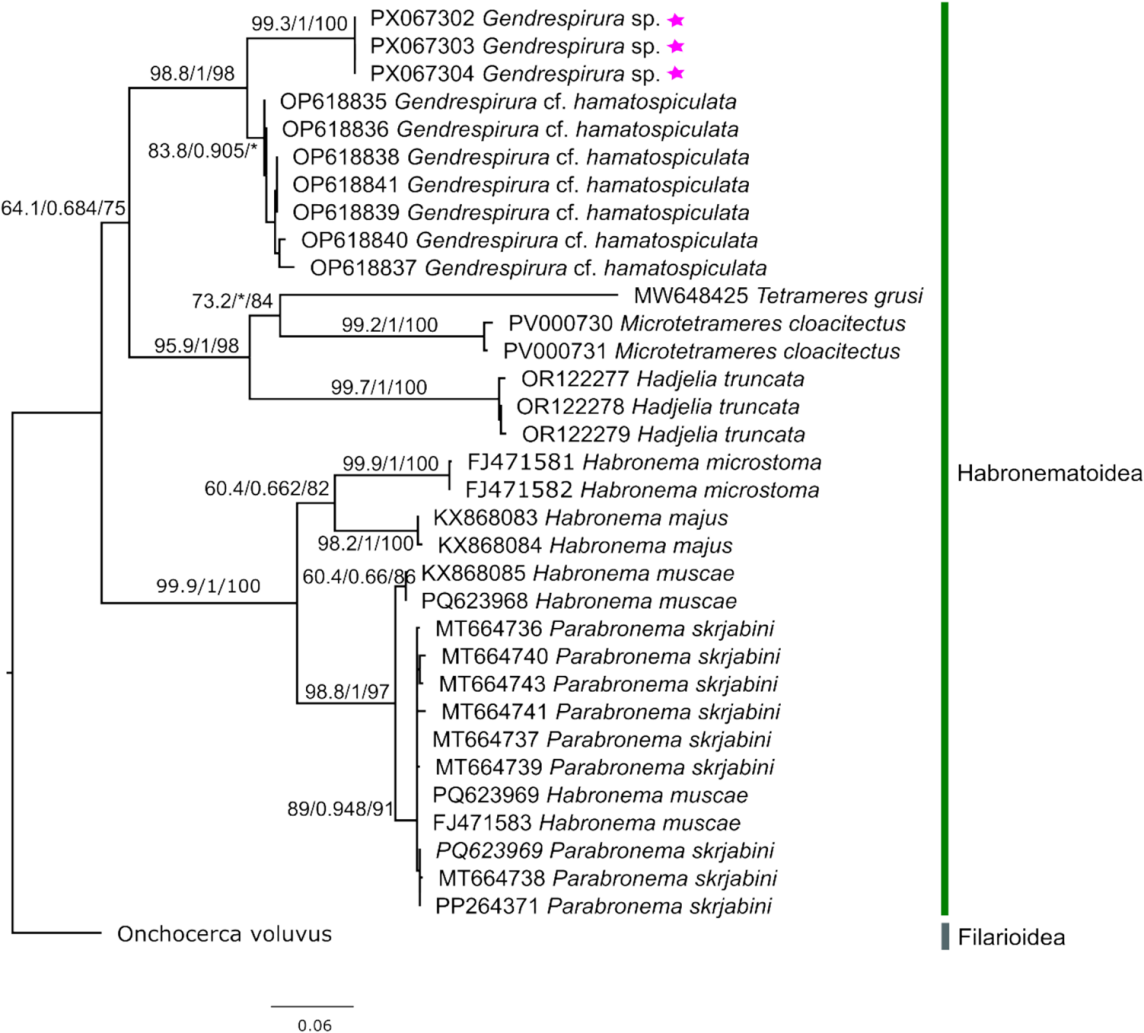
Maximum likelihood phylogenetic tree of a ~ 681 bp fragment of the mitochondrial COX1 gene from nematodes of Habronematoidea, including specimens recovered from a wild Sunda pangolin in Singapore (Genbank accessions: PX067302- PX067304). The tree (GTR + F + I + G4) was reconstructed in IQ-TREE v2.2.0 using three sequences generated in this study (indicated by stars), 31 reference sequences downloaded from GenBank, and one Filarioidea outgroup (*Onchocerca volvulus*) after alignment in MAFFT v7 with the G-INS-ialgorithm and trimming to a common region. Support was assessed using SH-aLRT, aBayes, and 1,000 ultrafast bootstrap replicates (Hoang et al. 2018). Node values are SH-aLRT/aBayes/bootstrap; asterisks (*) denote values below 60 or 0.6

Molecular analysis of three nematode specimens from case 2 revealed that they form a well-supported clade within the superfamily Habronematoidea, forming a sister clade to *Gendrespirura* cf. *hamatospiculata* (Fig. 3). A total of 35 COX1 sequences from Habronematoidea species, including *Gendrespirura* cf. *hamatospiculata* were analyzed alongside the Singapore specimens (Fig. 3). The resulting maximum likelihood phylogeny shows the Singapore sequences forming a distinct but strongly supported sister clade to *Gendrespirura* cf. *hamatospiculata*, with SH-aLRT/aBayes/bootstrap values of 98.8/1/98 at the branching node. The Singapore clade received strong support (99.3/1/100), while the *Gendrespirura* clade had moderate support (83.8/0.905/54). Pairwise evolutionary divergence between Singapore specimens and *Gendrespirura* cf. *hamatospiculata* clade was estimated at 0.0878 (SE = 0.0140) using the Kimura 2-parameter model with gamma-distributed rates (MEGA12, Kumar et al. 2024), across 681 aligned positions.

In addition to the primary COX1 analysis, supplementary phylogenies were constructed using 18S rRNA and a shorter non-overlapping COX1 fragment amplified using the primers of Li et al. (2024) (Figs. S1–S2). The 18S sequence showed 100% identity to a novel Spiruromorpha species from a pangolin in China (OR527425) and grouped in a well-supported clade (90.9/0.997/99). Similarly, the Li-primer COX1 sequences clustered with Spiruromorpha sp. (OR520278), with high node support (98.5/1/98). These analyses corroborate the placement of the Singapore specimens within the infraorder Spiruromorpha, however, due to their limited overlap and taxon representation, they did not resolve relationships at the genus level.

## Discussion

This is the first report identifying parasitic gastric disease caused by *Gendrespirura* sp. in wild Sunda pangolins in Singapore. Although these animals died from other un-related causes, the pathological findings associated with the parasite infection are significant and we cannot rule out potential subsequent morbidity due to gastric disease if the animals had survived. Locally extensive neutrophilic inflammation in case 1 was severe. However, inflammation in case 2 was minimal, showing more hyperkeratotic and cystic changes of the stratum corneum layer of the gastric mucosal epithelium instead. The disease severity difference in cases 1 and 2 is not immediately apparent but could be due to individual immunity, stress or parasitic life stages. From 2019 to 2024, 92 post-mortem examinations were performed on wild Sunda pangolins submitted to the Singapore Zoo, out of which 77 were in suitable condition for detailed examination, histopathology, and parasite retrieval. Although the prevalence of parasitic gastritis is low (2/77, 2.60%), continued long-term monitoring would benefit the local population. Gastric ulceration and hemorrhage in confiscated and rescued animals from the illegal wildlife trade have been reported (Wicker et al. 2020) but have not been observed in wild Sunda pangolins examined at the Singapore Zoo. Sunda pangolins intercepted by Hong Kong border authorities from illegal trafficking (Barton et al. 2022) showed a low prevalence of infection with the parasite identified as *Gendrespirura* cf. *zschokkei* (2/88, 2.27%). A novel Spiruromorpha species was identified from the small intestine in a Chinese pangolin (*Manis pentadactyla*) by molecular methods, but the clinical significance of this detection and histopathological examination was not reported (Li et al. 2024). Polypoid gastritis by *Gendrespirura* sp. infection in a Sunda pangolin from Malaysia was reported by Gardiner and Werner (1984). The findings from case 1 were very similar to those reported in Gardiner and Werner (1994), and based on size and the lack of reproductive organs, the authors suggest that the embedded larvae are that of *Gendrespirura* sp. Unfortunately, no parasite specimens from case 1 were available for molecular analysis and confirmation.

In case 2, while histopathological findings could only identify the parasites to the order of Rhabditida (Gardiner and Poynton 1999), microscopic findings of adult and egg specimens place them within the family Habronematidae. Overall body measurements of the specimens collected in this study matched those for *H. hamospiculatum* described by Hsü 1932 and those for *G.* cf. *zschokkei* described by Barton et al. 2022 (Table 2). Although reference molecular data for other *Gendrespirura* species remain limited, the three Singapore specimens consistently formed a well-supported clade with *Gendrespirura* cf. *hamatospiculata* in the COX1 phylogeny and are most likely members of the genus *Gendrespirura*. The reference sequences used here correspond to *G.* cf. *hamatospiculata* as deposited in GenBank by Barton et al. (2022), although in their publication the taxon was referred to as *G.* cf. *zschokkei*. This higher-level grouping received strong topological support (SH-aLRT/aBayes/bootstrap = 98.8/1/98), supporting the placement of the specimens within the family Habronematidae and the superfamily Habronematoidea. Within this clade, the Singapore specimens also formed a distinct and strongly supported subgroup (99.3/1/100), suggesting they may represent a geographically distinct lineage in Singapore, or potentially a more divergent form not currently represented among recognized *Gendrespirura* species. However, given the moderate support for the broader classification (64.1/0.684/75) and the high variability of the COX1 gene, additional molecular data from more *Gendrespirura* species and populations will be critical to clarify this relationship.

To complement the COX1 analysis, two additional fragments were analyzed using sequences amplified with Li et al. (2024) primers: a shorter COX1 fragment and 18S rRNA. These regions were analyzed separately due to minimal overlap with the Barton-COX1 fragment and sparse taxon representation in GenBank. Both fragments consistently placed the Singapore specimens in clades with high support alongside the Spiruromorpha sp. reported from a Chinese pangolin (Figs. S1, S2) (COX1: OR520278 and 18S rRNA: OR527425) and the 18S rRNA sequence was 100% identical to this taxon. Li et al.’s mitogenomic phylogeny placed their specimen in a clade near *Spirocerca lupi* (family Thelaziidae), but lacked *Gendrespirura* or other Habronematidae mitogenomes, limiting genus-level resolution. These additional markers corroborate placement within the infraorder Spiruromorpha and when interpreted together with the Barton-COX1 analysis, provide complementary evidence that the Spiruromorpha sp. reported by Li et al. (2024) is likely to represent a member of the genus *Gendrespirura*.

Other nematodes previously reported in the Sunda pangolin include *Ancylostoma* sp., *Brugia malayi*, *B. pahangi* and *Necator americanus* (Mohapatra et al. 2016; Wicker et al. 2020; Barton et al. 2022; Tuli et al. 2022). In other anteater species, *Physaloptera magnipapilla*, *Brevigraphidium dorsuarium*, *Graphidiops dissimilis* and other unknown trichostrongylids were detected in the stomachs of free-ranging giant anteaters (*Myrmecophaga tridactyla*) and lesser anteaters (*Tamandua tetradactyla*) in Brazil with gastric mucosal hyperplasia reported in one giant anteater associated with *G. dissimilis* infection (Arenales et al. 2020). Hyperplastic catarrhal gastritis caused by *P. magnipapilla* was reported in one wild giant anteater in Argentina (Lértora et al. 2016). Clinical signs included anorexia and ascites, with severe hypochromic microcytic anemia, hypoproteinemia resulting in death. Histopathological examination showed inflammatory infiltrates of plasma cells, lymphocytes, multinucleated giant cells and eosinophils at the sites of parasite attachment (Lértora et al. 2016).

## Conclusion

This research provides significant insights into the parasitic fauna of Sunda pangolins in Singapore, advancing our understanding of pangolin parasitology and the clinical implications of parasites on their health. Additionally, it raises questions about the significance of other parasites identified in pangolins and highlights the need for continuous active and passive surveillance of pathogens in this species. Such efforts would significantly deepen our knowledge of this understudied species and inform both in-situ and ex-situ conservation strategies.

## Supplementary Information

Below is the link to the electronic supplementary material.

Supplementary file1 (DOCX 318 KB)

Supplementary file2 (XLSX 19 KB)

## Data Availability

Sequences for nematodes retrieved from Sunda pangolins in Singapore are available in the NCBI GenBank database (https://www.ncbi.nlm.nih.gov/) under accession numbers PX067941–PX067943 for 18S rRNA and PX067302–PX067304 for COX1.
