# Supplementary material for: Parasitic gastritis in wild sunda pangolins (Manis Javanica), Singapore
Source: Parasitol Res. 2025 Oct 1;124(10):111. doi: 10.1007/s00436-025-08567-0 (PMC12488804; doi:10.1007/s00436-025-08567-0)
Supplement: Supplementary file 1 — Supplementary file1 (DOCX 318 KB) [file 436_2025_8567_MOESM1_ESM.docx]

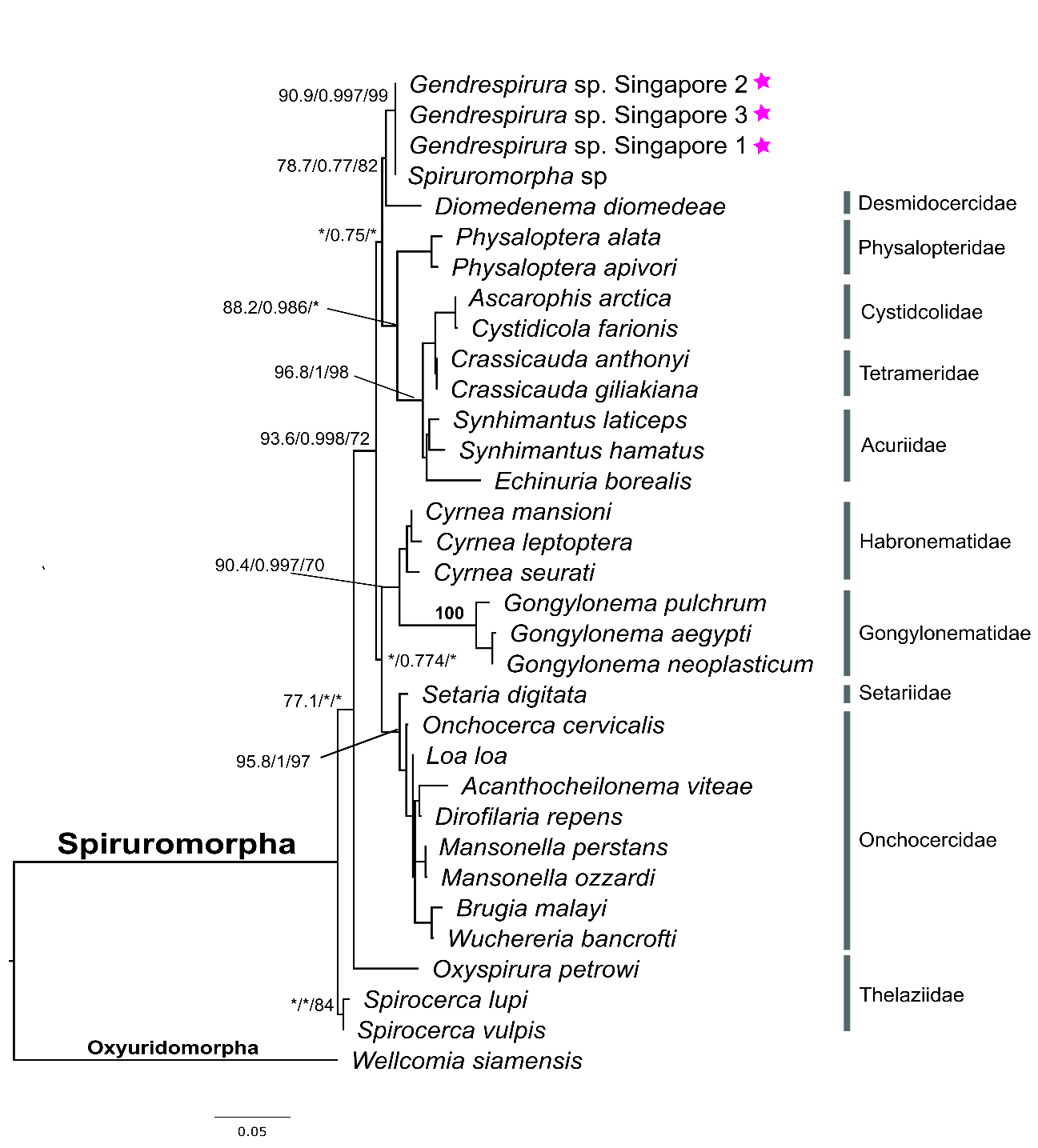


**Figure S1.**
Maximum likelihood phylogenetic tree based on a ~850 bp fragment of the 18S rRNA gene amplified with Li et al. (2024) primers. The Singapore specimens (marked with stars) clustered with high support alongside *Spiruromorpha* sp. (OR527425), to which they share 100% sequence identity. Reference sequences span multiple families within Spiruromorpha. Node support values represent SH-aLRT/aBayes/bootstrap; asterisks (*) indicate values <60 or 0.6. The tree was reconstructed using IQ-TREE under the best-fit model (GTR+F+I+G4) and visualised in FigTree v1.4.4.


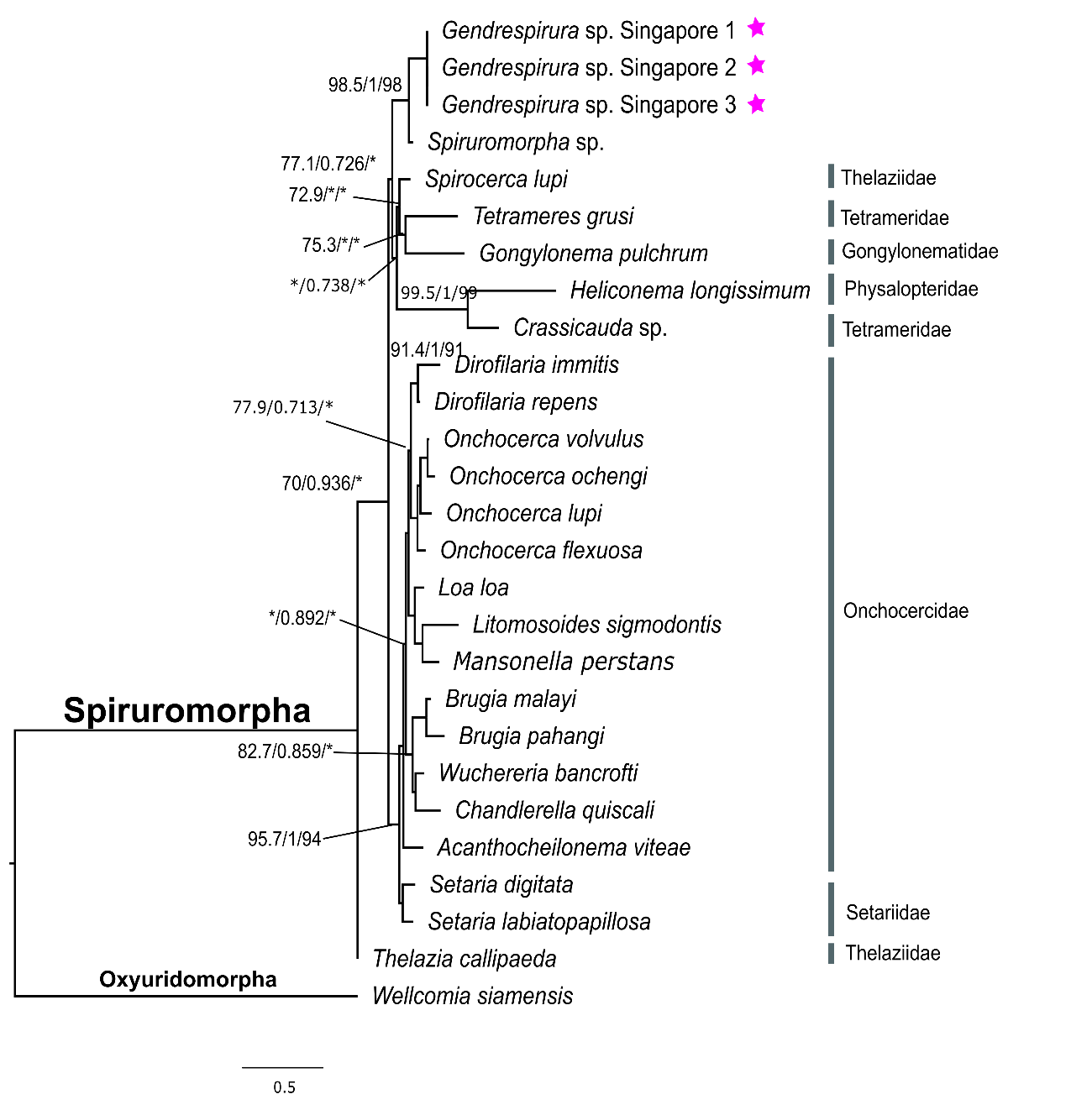


**Figure S2.**
Maximum likelihood phylogenetic tree based on a ~441 bp fragment of the mitochondrial COX1 gene amplified with Li et al. (2024) primers. The tree includes three Singapore nematode sequences (marked with stars) and reference sequences from across the infraorder Spiruromorpha, including *Spiruromorpha* sp. (OR520278). Node support values represent SH-aLRT/aBayes/bootstrap; asterisks (*) indicate values <60 or 0.6. The tree was reconstructed using IQ-TREE under the best-fit model (GTR+F+I+G4) and visualised with FigTree v1.4.4.
